# Progress in Renal Medicine

**Published:** 1901-01-05

**Authors:** 


### Progress in Renal Medicine.

**Pathology.**—J. B. Nash[1] gives an analysis of the work done by the remaining kidney tissue of three patients in whom some portion of the kidneys had been removed.

In the first patient one kidney had been taken away three years before, yet she was found to excrete on the average 1,520 cc. of urine, with 25 grammes of urea daily, which is above the normal for healthy persons with two kidneys. Indeed, she passed as much as 65 grammes of urea in a single day. A man with a kidney and a half showed 1,167 cc. and 24 grammes on the average at a date two years after operation. A third, a woman, who lost one kidney, showed directly after that operation an average of 898 cc. and 14 grammes, the water gradually increasing for the two months observed. This last case differs from that of the dogs reported by Rose Bradford, who found an increase of water and a normal excretion of urea, if at least one-third of the whole tissue was left in the animal. Though here then the water was actually diminished, Nash thinks the urea was not below the average for a woman recovering from a severe operation with two intact kidneys. Probably, then, we may say, if a third or more of the kidney weight is left after operation a patient can sustain a healthy life. Alessandri[2] examined the results of ligation of (*a*) the efferent vein, or (*b*) the renal artery, or (*c*) of nephrectomy, since in wounds and other states a surgeon may be compelled to choose one of these methods. If the vein was ligatured in animals, a collateral circulation was at last re-established and some part of the kidney recovered from the effects of the stasis and carried on the work. If the artery was tied in cats the whole kidney died, while in dogs it largely recovered after a collateral circulation was formed. In man there are so many collateral channels that recovery after ligature may be hoped for. The freezing point of urine, together with its quantity, may be taken as a test whether the kidneys are performing their work. Kovesi and Schultz[3] thus tested the urine excreted after drinking large quantities of fluid, both in healthy persons and in the subjects of nephritis. In parenchymatous disease, if advanced, the power to secrete diluted urine was much reduced, but in interstitial affections there was little failure. The question of an internal secretion by the kidney was first suggested by Brown-Sequard, who thought that uræmia might be due to the loss of this substance rather than to the failure of elimination of urea and similar products. Several authors found that animals whose kidneys had been removed lived longer if kidney juice or blood from the renal veins was injected into their circulation, and clinically it has often been noted that in suppression of urine true uræmia may be absent, presumably because the internal secretion is maintained. Chatin and Guinard,[4] however, find that in all cases in which renal venous blood was injected by them after nephrectomy death followed sooner than without it. Hence their results are opposed to the existence of an internal secretion.

**Ocular changes in Kidney Disease.**—W. O. Moore[5] estimates that 23 per cent. of kidney cases suffer from retinitis albuminurica, and complain of gradual failure of vision. In the exudative form, which occurs most often with parenchymatous nephritis, recovery of vision is possible. Indeed, in scarlatinal and puerperal cases complete repair of the ocular structures may take place. However, only 18 per cent. of the latter even recover their vision completely. Another affection, amaurosis, comes on suddenly in some uræmic patients with complete blindness, but may pass away as quickly. Death usually follows retinitis albuminurica in eighteen months, but puerperal patients and a few of the others may survive for long periods. W. Porter, in discussing this paper, thought that ocular changes in renal diseases chiefly occur where the patients have previously suffered from syphilis. He had found this conjunction almost invariably.

**Cystoscopy.**—G. Barling[6] gives a good deal of practical advice on the use of the cystoscope, which is of great value in determining the nature and situation of renal and bladder diseases. Complete asepsis is highly important, and in tubercular disease this is doubly so, where, indeed, all interference with the bladder is undesirable if it can be avoided. The bladder should usually be distended by the injection of a boric solution, and examinations should not be made when blood is flowing, since it obscures the view; yet we may need to examine at such a time, if the blood comes from a kidney, so as to observe it actually flowing from a ureter. It is discharged in jets from the peristaltic waves of the ureter, which look like the explosions of a miniature volcano. With much blood or pus it may be needful to wash out the bladder several times to get a clear view. A blood clot is liable to be mistaken for a tumour, but is easily movable. Again, in withdrawing the cystoscope a bright red mass shows up where the urethra joins the bladder. This, too, may be mistaken for a tumour.

**Movable Kidneys.**—These sometimes cause few symptoms, or they may render life intolerable. C. Mansell Moullin[7] defines them as those which fail to reascend on tranquil expiration when the patient is standing upright. Thus the extent to which the kidney descends on inspiration is no test of abnormal mobility. Neither the surrounding fat, nor the fascia, nor the peritoneum hold the kidney in place when the abdominal pressure is taken away. Sometimes the lumbar recess, in which the kidney is lodged, is deficient. In many cases of movable right kidney there is flattening of the right lumbar region, due to lateral deviation of the spine and rotation forwards of the transverse processes on that side making the recess or bed of the kidney shallower. Hence the frequency of movable kidneys on the right side. Besides the pain produced by movement, he thinks the shocks tend to cause calculous and tuberculous disease, rather than that these diseases lead to movable kidney. He advises belts for the milder cases and nephrorraphy for the severer, including all those where there is definite flattening of the corresponding lumbar region, to prevent further damage.

**Cystic Kidney.**—G. L. Peabody[8] reports an instance where both kidneys (weighing nearly 15 oz. each) and the liver were affected. In this case the patient was a woman of forty-four, and had complained of weakness for six months, and at last some dyspnœa and œdema. Before death she showed slight jaundice, pus and albumen in the urine with no casts. He took the view of their congenital origin, but gave no arguments for this view. Norman Dalton[9] showed a woman of thirty-five with large cystic kidneys forming elastic swellings in the lumbar regions. The urine was pale, 1012, sometimes abundant, sometimes giving a trace of albumen. The urea was only half the normal, but there was high-tension pulse. In the discussion the absence of cardio-vascular changes in some cases was noted.

**Effects of Anæsthetics.**—W. H. Thompson[10] in experiments on dogs found that (*a*) under ether there was generally increased diuresis, continuing some time afterwards; (*b*) under chloroform there was diminished diuresis, and subsequent increase; (*c*) under A.C.E. the results were variable, and under ether-chloroform mixture there was little change. Hence this method is the best for operations on the kidney. Generally speaking, the chlorides are diminished, but there seems no reason for thinking that albuminuria is caused by any anæsthetic. Thompson and Kemp elsewhere had reported a specific effect of ether in causing a fall and even suppression of urine and the appearance of albumen. Buxton and Levy[11] deny that this is constant, and think it occurs only after excessive amounts of ether. In 37 cases of surgical operations under ether they found some diminution of the diuresis, but little or no increase of albumen. The diminution may have been due to other causes, *e.g.* shock and vomiting, and the specific gravity was actually increased. Therefore if any injury to the kidney from ether occurs, they believe it is only when excessive amounts are given.

**Complications.**—The *Bacillus aërogenes capsulatus* sometimes gains entrance to the body by the urinary passages, and infects them primarily, though it usually enters by wounds or the intestinal canal. Welch[12] mentions their occurrence in an old stricture case where they had obtained access to the kidney and the general circulation. He has collected 46 cases of emphysematous gangrene, or gas—containing abscesses due to it. When free incisions or amputations can be performed recovery is not impossible. Swelling of the eye-lids with occasional presence of albuminuria in children is discussed by Theodore Fisher.[13] He gives instances where this occurred, and remarks that their general health was fair, though they were listless. Other children show similar swellings with no albumen, and he would regard both the swelling and occasional albuminuria as not due to kidney disease. Brill and Libman[14] think that chronic nephritis, and especially interstitial, is commoner in children than we usually suppose. They report a fresh case of interstitial nephritis, and refer to 30 others collected by Heubner. They draw attention to the latency of some of these instances. Their patient was fourteen years of age, showed marked vascular changes, and the disease existed in other members of her family.

**Urology.**—Surveyor reports[15] a recurrent erysipelatous swelling, with vesicles on the face and hands, which was accompanied by urine giving a pink reaction with caustic soda. The substance which causes this change appears to exist in other diseases, but its composition is unknown, though it is certainly not a peptone. Bence-Jones's albumen, which is found in myologenic sarcoma and perhaps in myxœdema also, is declared by Magnus Levy[16] not to be albumose. It is sometimes soluble at 100°C., and does not seem to be formed in the tumours. Indeed these special growths are not always accompanied by this constituent of the urine. Oxalic acid in health is, according to the investigations of Helen Baldwin,[17] due to that ingested in the food; but in some diseases, especially where HCl is absent from the stomach and fermentative changes take place, oxalic acid is actually formed in the body. The symptoms commonly attributed to the oxalic acid diathesis are due chiefly to the action of other bodies liberated by the fermentation, though the oxalates themselves cause the irritation of the urinary tract. The ordinary methods of estimating the oxalates in the urine are apt to lead to serious errors unless the greatest precautions are taken. W. O. Moor[18] argues that urine contains a liquid organic substance to which most of its properties are due, and which he calls "ureine." To it he attributes the production of uræmia, ammoniacal fermentation, &c. To obtain it he evaporates the urine at a moderate temperature, and removes the phosphates, chlorides, &c. He claims that it is an aromatic alcohol of the specific gravity 1·270, and possessing a characteristic odour.

[1] Lancet, June 30. [2] Med. Rec., April 14. [3] Med. Rec., June 30. [4] Lancet, May 8. [5] Post Grad., July. [6] Birmingham Med. Rec., July. [8] Lancet, May 5. [8] Med. Rec., March 17. [9] Lancet, Nov. 3. [10] Brit. Med. Jour., Sept. 2. [11] Soc. cit. [12] Lancet, Sept. 8. [13] Brit. Med. Jour., April 14. [14] Amer. Jour. Med. Sci., April. [15] Lancet, Aug. 11. [16] Lancet, May 26. [17] Med. Rec., Oct. 13. [18] Med. Rec., Sept. 1.

